# 2,2-Bis(1*H*-indol-3-yl)indolin-3-one

**DOI:** 10.1107/S1600536809017553

**Published:** 2009-05-20

**Authors:** Zhao-Hao Li, Jing Xu, Wen-Liang Wu, Wei-Ping Su

**Affiliations:** a Graduate School, The Chinese Academy of Sciences, Beijing 100039, People’s Republic of China; b The State Key Laboratory of Structural Chemistry, Fujian Institute of Research on the Structure of Matter, The Chinese Academy of Sciences, Fuzhou, Fujian 350002, People’s Republic of China

## Abstract

In the title mol­ecule, C_24_H_17_N_3_O, the mean plane of the indolone ring forms dihedral angles of 112.0 (1) and 103.1 (1)° with the planes of the two indole rings. The dihedral angle between the mean planes of the two indole rings is 63.5 (1)°. In the crystal structure, mol­ecules are linked *via* inter­molecular N—H⋯O hydrogen bonds, forming a two-dimensional network parallel to the *ab* plane.

## Related literature

For the applications of indole derivatives, see: Ramesh *et al.* (2009 ▶). For the isolation of the title compound as a natural product, see: Ganachaud *et al.* (2008 ▶); Stull *et al.* (1995 ▶).
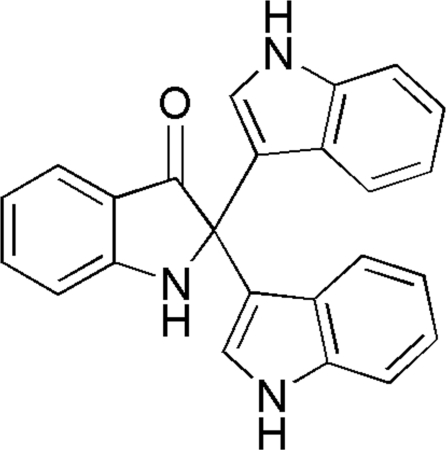


## Experimental

### 

#### Crystal data

C_24_H_17_N_3_O
*M* *_r_* = 363.41
Monoclinic, 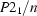

*a* = 10.559 (4) Å
*b* = 8.931 (3) Å
*c* = 19.899 (7) Å
β = 98.480 (6)°
*V* = 1856.1 (11) Å^3^
*Z* = 4
Mo *K*α radiation
μ = 0.08 mm^−1^
*T* = 293 K
0.40 × 0.35 × 0.15 mm

#### Data collection

Rigaku Mercury CCD diffractometer
Absorption correction: multi-scan (*CrystalClear*; Rigaku/MSC, 2005 ▶) *T*_min_ = 0.968, *T*_max_ = 0.988
14004 measured reflections
4245 independent reflections
3606 reflections with *I* > 2σ(*I*)
*R*_int_ = 0.024

#### Refinement

*R*[*F* ^2^ > 2σ(*F* ^2^)] = 0.048
*wR*(*F* ^2^) = 0.118
*S* = 1.06
4245 reflections
253 parameters
H-atom parameters constrained
Δρ_max_ = 0.21 e Å^−3^
Δρ_min_ = −0.19 e Å^−3^

### 

Data collection: *CrystalClear* (Rigaku/MSC, 2005 ▶); cell refinement: *CrystalClear*; data reduction: *CrystalClear*; program(s) used to solve structure: *SHELXS97* (Sheldrick, 2008 ▶); program(s) used to refine structure: *SHELXL97* (Sheldrick, 2008 ▶); molecular graphics: *DIAMOND* (Brandenburg, 1999 ▶); software used to prepare material for publication: *SHELXL97*.

## Supplementary Material

Crystal structure: contains datablocks I, global. DOI: 10.1107/S1600536809017553/lh2811sup1.cif

Structure factors: contains datablocks I. DOI: 10.1107/S1600536809017553/lh2811Isup2.hkl

Additional supplementary materials:  crystallographic information; 3D view; checkCIF report

## Figures and Tables

**Table 1:** Hydrogen-bond geometry (Å, °)

*D*—H⋯*A*	*D*—H	H⋯*A*	*D*⋯*A*	*D*—H⋯*A*
N2—H2*B*⋯O1^i^	0.86	2.12	2.9412 (17)	159
N3—H3*B*⋯O1^ii^	0.86	2.18	2.9830 (16)	156

Symmetry codes: (i) 
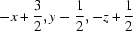
; (ii) 
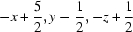
.

**Fig. 1.**
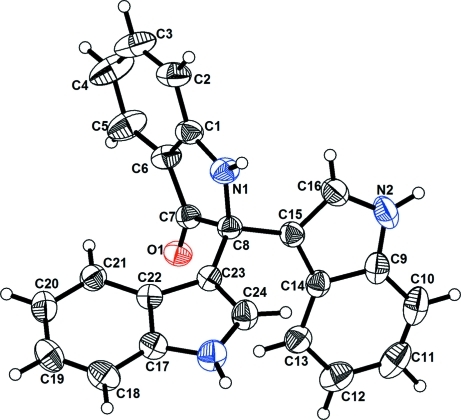
The molecular structure of (I), showing 30% probability displacement ellopsoids. H atoms are shown as small spheres.

**Fig. 2.**
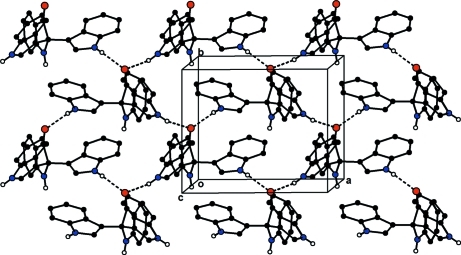
Part of the crystal structure of (I) showing the hydrogen bonded layers parallel to the ab plane. Intermolecular hydrogen bonds are shown as dashed lines and H atoms not involved in H-bonding have been omitted.

## supplementary crystallographic information

### Comment

Indole derivatives are used as bioactive drugs and they exibit anti-allergic, central nervous system depressant and muscle relaxant properties (Ramesh *et al.*, 2009). The title compound is a natural product that has been isolated from bacterial sources (Stull *et al.*., 1995; Ganachaud *et al.*., 2008). Recently, we found different indole derivatives could be formed by oxidation solely on the basis of the reaction solvent and temperature. As part of our studies, we report herein the synthesis and crystal structure of the title compound (I) (Fig. 1).

The title compound is a trimeric condensation product of indole through the formation of a quaternary carbon (C8) centre. The indolone ring forms dihedral angle of 112.0 (1)° and 103.1 (1)°, respectively, with the two indole rings (C9/C10/C11/C12/C13/C14/C15/C16/N2) and (C17/C18/C19/C20/C21/C22/C23/C24/N3). The mean planes of the two indole form a dihedral angle of 63.5 (1)°. In the crystal structure, the carbonyl atom O1 acts as a bifurcated acceptor for the N-H groups of atoms  N2 and N3 to form a two-dimensional network parallel to the *ab* plane (Table 1, Fig. 2).

### Experimental

The title compound was obtained from a mixture of 1*H*-indole (78 mg) with *^t^*BuCOOH (0.18 ml) and H_3_PMo_12_O_14_ (9 mg) in toluene (1 ml) and CH_3_COOH (1 ml) under a nitrogen atmosphere at room temperature for 24 h. The crude product was isolated and purified by silica gel columnchromatography. Yellow prism-shaped crystals of (I) suitable for X-ray diffraction were grown by slow evaporation of a dichloromethane solution at room temperature.

### Refinement

H atoms were positioned geometrically and allowed to ride on their parent atoms, with C—H = 0.93 Å;  N—H = 0.86 Å and *U*_iso_(H) = 1.2Ueq(C,N).

### Crystal data

**Table:**

C_24_H_17_N_3_O	*F*(000) = 760
*M**_r_* = 363.41	*D*_x_ = 1.300 Mg m^−^^3^
Monoclinic, *P*2_1_/*n*	Mo *K*α radiation, λ = 0.71073 Å
Hall symbol: -P 2yn	Cell parameters from 4234 reflections
*a* = 10.559 (4) Å	θ = 3.0–27.5°
*b* = 8.931 (3) Å	µ = 0.08 mm^−^^1^
*c* = 19.899 (7) Å	*T* = 293 K
β = 98.480 (6)°	Prism, yellow
*V* = 1856.1 (11) Å^3^	0.40 × 0.35 × 0.15 mm
*Z* = 4	

### Data collection

**Table:**

Rigaku Mercury CCD diffractometer	4245 independent reflections
Radiation source: fine-focus sealed tube	3606 reflections with *I* > 2σ(*I*)
graphite	*R*_int_ = 0.024
Detector resolution: 14.6306 pixels mm^-1^	θ_max_ = 27.5°, θ_min_ = 2.1°
CCD_Profile_fitting scans	*h* = −11→13
Absorption correction: multi-scan (*CrystalClear*; Rigaku/MSC, 2005)	*k* = −11→11
*T*_min_ = 0.968, *T*_max_ = 0.988	*l* = −22→25
14004 measured reflections	

### Refinement

**Table:**

Refinement on *F*^2^	Primary atom site location: structure-invariant direct methods
Least-squares matrix: full	Secondary atom site location: difference Fourier map
*R*[*F*^2^ > 2σ(*F*^2^)] = 0.048	Hydrogen site location: inferred from neighbouring sites
*wR*(*F*^2^) = 0.118	H-atom parameters constrained
*S* = 1.06	*w* = 1/[σ^2^(*F*_o_^2^) + (0.0513*P*)^2^ + 0.4603*P*] where *P* = (*F*_o_^2^ + 2*F*_c_^2^)/3
4245 reflections	(Δ/σ)_max_ < 0.001
253 parameters	Δρ_max_ = 0.21 e Å^−^^3^
0 restraints	Δρ_min_ = −0.19 e Å^−^^3^

### Special details

**Table:**

Geometry. All e.s.d.'s (except the e.s.d. in the dihedral angle between two l.s. planes) are estimated using the full covariance matrix. The cell e.s.d.'s are taken into account individually in the estimation of e.s.d.'s in distances, angles and torsion angles; correlations between e.s.d.'s in cell parameters are only used when they are defined by crystal symmetry. An approximate (isotropic) treatment of cell e.s.d.'s is used for estimating e.s.d.'s involving l.s. planes.
Refinement. Refinement of *F*^2^ against ALL reflections. The weighted *R*-factor *wR* and goodness of fit *S* are based on *F*^2^, conventional *R*-factors *R* are based on *F*, with *F* set to zero for negative *F*^2^. The threshold expression of *F*^2^ > σ(*F*^2^) is used only for calculating *R*-factors(gt) *etc*. and is not relevant to the choice of reflections for refinement. *R*-factors based on *F*^2^ are statistically about twice as large as those based on *F*, and *R*- factors based on ALL data will be even larger.

### Fractional atomic coordinates and isotropic or equivalent isotropic displacement parameters (Å^2^)

**Table:**

	*x*	*y*	*z*	*U*_iso_*/*U*_eq_	
O1	0.96565 (9)	0.43252 (10)	0.15911 (5)	0.0369 (2)	
N1	0.95403 (12)	0.04726 (13)	0.11858 (6)	0.0406 (3)	
H1A	0.9668	−0.0474	0.1239	0.049*	
N2	0.75509 (12)	0.05077 (15)	0.28328 (7)	0.0490 (3)	
H2B	0.6914	−0.0017	0.2923	0.059*	
N3	1.31856 (12)	0.10180 (15)	0.26178 (6)	0.0471 (3)	
H3B	1.3724	0.0659	0.2945	0.056*	
C1	0.89773 (13)	0.11138 (16)	0.05986 (7)	0.0374 (3)	
C2	0.85094 (14)	0.0420 (2)	−0.00189 (8)	0.0483 (4)	
H2A	0.8532	−0.0615	−0.0065	0.058*	
C3	0.80181 (17)	0.1314 (2)	−0.05518 (8)	0.0635 (5)	
H3A	0.7706	0.0868	−0.0966	0.076*	
C4	0.7967 (2)	0.2867 (3)	−0.04991 (9)	0.0746 (6)	
H4A	0.7630	0.3437	−0.0874	0.090*	
C5	0.84151 (19)	0.3558 (2)	0.01064 (8)	0.0604 (5)	
H5A	0.8383	0.4595	0.0147	0.072*	
C6	0.89185 (13)	0.26719 (17)	0.06585 (7)	0.0392 (3)	
C7	0.94976 (12)	0.30732 (14)	0.13383 (6)	0.0315 (3)	
C8	0.99057 (13)	0.15946 (14)	0.17220 (6)	0.0321 (3)	
C9	0.81798 (14)	0.15783 (17)	0.32497 (7)	0.0417 (3)	
C10	0.79721 (18)	0.2065 (2)	0.38885 (8)	0.0557 (4)	
H10A	0.7324	0.1655	0.4100	0.067*	
C11	0.8753 (2)	0.3166 (2)	0.41938 (8)	0.0616 (5)	
H11A	0.8635	0.3509	0.4621	0.074*	
C12	0.97253 (17)	0.3786 (2)	0.38756 (8)	0.0550 (4)	
H12A	1.0236	0.4541	0.4093	0.066*	
C13	0.99417 (15)	0.32982 (17)	0.32449 (7)	0.0424 (3)	
H13A	1.0597	0.3712	0.3040	0.051*	
C14	0.91605 (13)	0.21709 (15)	0.29179 (7)	0.0351 (3)	
C15	0.90865 (13)	0.13904 (15)	0.22798 (7)	0.0342 (3)	
C16	0.80919 (14)	0.04053 (17)	0.22537 (8)	0.0432 (3)	
H16A	0.7823	−0.0240	0.1894	0.052*	
C17	1.34968 (13)	0.18478 (15)	0.20847 (7)	0.0380 (3)	
C18	1.46884 (15)	0.23042 (18)	0.19416 (9)	0.0491 (4)	
H18A	1.5436	0.2065	0.2230	0.059*	
C19	1.47219 (15)	0.31203 (18)	0.13592 (9)	0.0511 (4)	
H19A	1.5507	0.3436	0.1251	0.061*	
C20	1.36003 (15)	0.34840 (18)	0.09265 (8)	0.0461 (4)	
H20A	1.3651	0.4030	0.0533	0.055*	
C21	1.24194 (14)	0.30469 (15)	0.10722 (7)	0.0380 (3)	
H21A	1.1679	0.3304	0.0782	0.046*	
C22	1.23417 (12)	0.22113 (14)	0.16619 (6)	0.0322 (3)	
C23	1.13200 (12)	0.15690 (14)	0.19763 (6)	0.0326 (3)	
C24	1.18882 (14)	0.08515 (16)	0.25473 (7)	0.0412 (3)	
H24A	1.1454	0.0325	0.2846	0.049*	

### Atomic displacement parameters (Å^2^)

**Table:**

	*U*^11^	*U*^22^	*U*^33^	*U*^12^	*U*^13^	*U*^23^
O1	0.0351 (5)	0.0344 (5)	0.0407 (5)	0.0007 (4)	0.0040 (4)	−0.0052 (4)
N1	0.0497 (7)	0.0320 (6)	0.0391 (6)	−0.0014 (5)	0.0029 (5)	−0.0058 (5)
N2	0.0361 (7)	0.0527 (8)	0.0603 (8)	−0.0086 (6)	0.0138 (6)	0.0105 (6)
N3	0.0361 (7)	0.0560 (8)	0.0472 (7)	0.0108 (6)	−0.0002 (5)	0.0156 (6)
C1	0.0288 (7)	0.0464 (8)	0.0367 (7)	−0.0010 (6)	0.0041 (5)	−0.0075 (6)
C2	0.0377 (8)	0.0583 (9)	0.0474 (8)	−0.0003 (7)	0.0016 (6)	−0.0203 (7)
C3	0.0549 (11)	0.0890 (14)	0.0415 (9)	0.0140 (10)	−0.0100 (8)	−0.0235 (9)
C4	0.0916 (16)	0.0844 (14)	0.0395 (9)	0.0287 (12)	−0.0181 (9)	−0.0053 (9)
C5	0.0750 (12)	0.0580 (10)	0.0423 (8)	0.0195 (9)	−0.0110 (8)	−0.0010 (7)
C6	0.0353 (7)	0.0455 (8)	0.0347 (7)	0.0055 (6)	−0.0015 (6)	−0.0053 (6)
C7	0.0252 (6)	0.0364 (7)	0.0330 (6)	0.0018 (5)	0.0048 (5)	−0.0025 (5)
C8	0.0327 (7)	0.0322 (6)	0.0311 (6)	−0.0015 (5)	0.0034 (5)	−0.0032 (5)
C9	0.0367 (8)	0.0463 (8)	0.0437 (8)	0.0066 (6)	0.0114 (6)	0.0125 (6)
C10	0.0577 (10)	0.0660 (11)	0.0486 (9)	0.0120 (9)	0.0249 (8)	0.0170 (8)
C11	0.0735 (13)	0.0775 (13)	0.0365 (8)	0.0178 (10)	0.0174 (8)	0.0021 (8)
C12	0.0601 (11)	0.0623 (10)	0.0419 (8)	0.0047 (8)	0.0051 (7)	−0.0090 (7)
C13	0.0425 (8)	0.0485 (8)	0.0366 (7)	−0.0009 (6)	0.0067 (6)	−0.0014 (6)
C14	0.0327 (7)	0.0391 (7)	0.0338 (6)	0.0038 (5)	0.0058 (5)	0.0054 (5)
C15	0.0309 (7)	0.0353 (7)	0.0359 (7)	−0.0004 (5)	0.0039 (5)	0.0026 (5)
C16	0.0367 (8)	0.0436 (8)	0.0486 (8)	−0.0051 (6)	0.0044 (6)	0.0023 (6)
C17	0.0338 (7)	0.0366 (7)	0.0430 (7)	0.0063 (5)	0.0037 (6)	0.0013 (6)
C18	0.0297 (7)	0.0524 (9)	0.0640 (10)	0.0058 (6)	0.0030 (7)	0.0001 (8)
C19	0.0359 (8)	0.0515 (9)	0.0686 (11)	−0.0027 (7)	0.0172 (7)	−0.0014 (8)
C20	0.0472 (9)	0.0473 (8)	0.0463 (8)	−0.0034 (7)	0.0154 (7)	0.0031 (7)
C21	0.0371 (7)	0.0407 (7)	0.0359 (7)	0.0006 (6)	0.0049 (6)	0.0006 (6)
C22	0.0313 (7)	0.0308 (6)	0.0346 (6)	0.0036 (5)	0.0051 (5)	−0.0022 (5)
C23	0.0319 (7)	0.0327 (6)	0.0334 (6)	0.0029 (5)	0.0056 (5)	0.0001 (5)
C24	0.0376 (8)	0.0436 (8)	0.0428 (8)	0.0054 (6)	0.0070 (6)	0.0096 (6)

### Geometric parameters (Å, °)

**Table:**

O1—C7	1.2274 (16)	C9—C14	1.411 (2)
N1—C1	1.3571 (18)	C10—C11	1.367 (3)
N1—C8	1.4731 (16)	C10—H10A	0.9300
N1—H1A	0.8600	C11—C12	1.398 (3)
N2—C16	1.363 (2)	C11—H11A	0.9300
N2—C9	1.371 (2)	C12—C13	1.379 (2)
N2—H2B	0.8600	C12—H12A	0.9300
N3—C24	1.3645 (19)	C13—C14	1.400 (2)
N3—C17	1.3731 (19)	C13—H13A	0.9300
N3—H3B	0.8600	C14—C15	1.4405 (19)
C1—C6	1.399 (2)	C15—C16	1.3652 (19)
C1—C2	1.3997 (19)	C16—H16A	0.9300
C2—C3	1.367 (3)	C17—C18	1.391 (2)
C2—H2A	0.9300	C17—C22	1.4136 (19)
C3—C4	1.392 (3)	C18—C19	1.374 (2)
C3—H3A	0.9300	C18—H18A	0.9300
C4—C5	1.374 (2)	C19—C20	1.396 (2)
C4—H4A	0.9300	C19—H19A	0.9300
C5—C6	1.394 (2)	C20—C21	1.378 (2)
C5—H5A	0.9300	C20—H20A	0.9300
C6—C7	1.4451 (18)	C21—C22	1.4031 (19)
C7—C8	1.5542 (18)	C21—H21A	0.9300
C8—C23	1.5046 (19)	C22—C23	1.4434 (18)
C8—C15	1.5155 (19)	C23—C24	1.3640 (19)
C9—C10	1.391 (2)	C24—H24A	0.9300
			
C1—N1—C8	111.79 (11)	C10—C11—C12	121.26 (16)
C1—N1—H1A	124.1	C10—C11—H11A	119.4
C8—N1—H1A	124.1	C12—C11—H11A	119.4
C16—N2—C9	109.42 (12)	C13—C12—C11	121.24 (17)
C16—N2—H2B	125.3	C13—C12—H12A	119.4
C9—N2—H2B	125.3	C11—C12—H12A	119.4
C24—N3—C17	109.29 (12)	C12—C13—C14	119.00 (15)
C24—N3—H3B	125.4	C12—C13—H13A	120.5
C17—N3—H3B	125.4	C14—C13—H13A	120.5
N1—C1—C6	111.45 (12)	C13—C14—C9	118.37 (13)
N1—C1—C2	128.48 (14)	C13—C14—C15	135.19 (13)
C6—C1—C2	120.06 (14)	C9—C14—C15	106.43 (13)
C3—C2—C1	117.83 (16)	C16—C15—C14	106.67 (12)
C3—C2—H2A	121.1	C16—C15—C8	124.66 (13)
C1—C2—H2A	121.1	C14—C15—C8	128.66 (12)
C2—C3—C4	122.53 (15)	N2—C16—C15	109.91 (14)
C2—C3—H3A	118.7	N2—C16—H16A	125.0
C4—C3—H3A	118.7	C15—C16—H16A	125.0
C5—C4—C3	120.11 (17)	N3—C17—C18	130.01 (13)
C5—C4—H4A	119.9	N3—C17—C22	107.46 (12)
C3—C4—H4A	119.9	C18—C17—C22	122.53 (14)
C4—C5—C6	118.52 (17)	C19—C18—C17	117.69 (14)
C4—C5—H5A	120.7	C19—C18—H18A	121.2
C6—C5—H5A	120.7	C17—C18—H18A	121.2
C5—C6—C1	120.95 (14)	C18—C19—C20	121.24 (14)
C5—C6—C7	131.01 (14)	C18—C19—H19A	119.4
C1—C6—C7	107.97 (12)	C20—C19—H19A	119.4
O1—C7—C6	128.53 (13)	C21—C20—C19	121.07 (14)
O1—C7—C8	124.16 (12)	C21—C20—H20A	119.5
C6—C7—C8	107.31 (11)	C19—C20—H20A	119.5
N1—C8—C23	111.98 (11)	C20—C21—C22	119.54 (13)
N1—C8—C15	109.37 (11)	C20—C21—H21A	120.2
C23—C8—C15	113.41 (11)	C22—C21—H21A	120.2
N1—C8—C7	101.43 (10)	C21—C22—C17	117.92 (12)
C23—C8—C7	111.57 (10)	C21—C22—C23	135.52 (12)
C15—C8—C7	108.37 (11)	C17—C22—C23	106.56 (12)
N2—C9—C10	130.02 (15)	C24—C23—C22	106.43 (12)
N2—C9—C14	107.57 (13)	C24—C23—C8	125.45 (12)
C10—C9—C14	122.41 (15)	C22—C23—C8	128.05 (11)
C11—C10—C9	117.71 (15)	C23—C24—N3	110.25 (13)
C11—C10—H10A	121.1	C23—C24—H24A	124.9
C9—C10—H10A	121.1	N3—C24—H24A	124.9

### Hydrogen-bond geometry (Å, °)

**Table:**

*D*—H···*A*	*D*—H	H···*A*	*D*···*A*	*D*—H···*A*
N2—H2B···O1^i^	0.86	2.12	2.9412 (17)	159
N3—H3B···O1^ii^	0.86	2.18	2.9830 (16)	156

Symmetry codes: (i) −*x*+3/2, *y*−1/2, −*z*+1/2; (ii) −*x*+5/2, *y*−1/2, −*z*+1/2.
